# Augmentation Rhinoplasty in Cleft Lip Nasal Deformity: Preliminary Patients' Perspective

**DOI:** 10.1155/2014/202560

**Published:** 2014-09-01

**Authors:** William H. C. Tiong, Mohd Ali Mat Zain, Normala Hj Basiron

**Affiliations:** Department of Plastic and Reconstructive Surgery, Hospital Kuala Lumpur, Jalan Pahang, 50586 Kuala Lumpur, Malaysia

## Abstract

The correction of cleft lip nasal deformity is challenging and there have been numerous methods described in the literature with little demonstrated technical superiority of one over another. The common clinical issues associated with cleft lip nasal deformity are its lack of symmetry, alar collapse on the affected side, obtuse nasal labial angle, short nasal length, loss of tip definition, and altered columella show among others. We carried out augmentation of cleft lip rhinoplasties with rib graft in 16 patients over the one-year study period. Each of these patients was reviewed and given questionnaire before and after surgery to evaluate their response on the outcome to the approach. Preoperatively, nasal asymmetry is the main complaint (14/16, 87.5%) among our series of patients. Postoperatively, 12 (75%) patients out of the 16 reported significant improvement in their nasal symmetry with the other four marginal. All patients reported excellent nasal projection postoperatively with good nasal tip definition. Our series of patients reported overall good satisfaction outcome and will recommend this procedure to other patients with cleft lip nasal deformity. In conclusion, augmentation of cleft lip rhinoplasty can be employed to achieve perceivable and satisfactory outcome in patients with cleft lip nasal deformity.

## 1. Introduction 

The nose is one of the most visible organs on the face and its appearance contributes enormously to facial aesthetics [1]. Nasal deformity associated with cleft lip has been viewed as one of the most challenging reconstructive problems in rhinoplasty. The complexity of cleft lip rhinoplasty is demonstrated by the abundance of technique that is available for its correction [2]. Yet, there is no conclusively superior technique among those that were described to date.

The common clinical features associated with cleft lip nasal deformity are its lack of symmetry, alar collapse on the affected side, short nasal length, loss of tip definition, obtuse nasal labial angle, and altered columella show among others [3]. Despite the numerous above features, the typical nose of cleft lip nasal deformity can be summarized as having an asymmetrical, flat dorsum, broad tip, and wide alar base on the cleft side [4, 5]. The horizontally oriented wide nostril on the cleft side is one of the major stigmas of the cleft lip-associated nasal deformity [6].

There have been many anthropometric studies on normal nasal parameter and it is widely accepted that the shape and dimension of nose vary according to different racial and ethnic profiles [7, 8]. Despite numerous descriptions and classifications of Oriental nose, they can be similarly summarized as having a bulbous nasal tip and broad alar bases with lack of tip projection. In a study by Aung et al., it was found that most Oriental patients prefer to have a higher nasal dorsum, increased nasal tip projection, and less flaring of the alar bases [9]. This same, desired nasal feature is also requested among patients with cleft lip nasal deformity [10]. The demand for prominent and narrow nasal profile makes augmentation of the dorsum and tip of nose among the most commonly performed aesthetic rhinoplasty procedures among Orientals [11]. The use of L-shaped nasal strut implant, consisting of a dorsal nasal onlay graft and columellar strut, is a well-established technique in aesthetic Oriental rhinoplasty for combined augmentation of the dorsum and tip of nose, respectively [11]. Yonehara et al. had shown that the appearance of a cleft lip-associated nose can be similarly improved with a well-positioned cantilevered bone graft [10].

There are various materials that can be used to augment nasal dorsum [12–15]. Augmentation of dorsum of the nose can be achieved using alloplastic materials, bone, or cartilage. Although various types of alloplastic materials have been used for dorsal augmentation, they are hampered with long-term complications that make them unattractive for our long-term cleft lip nasal deformity correction [12–15]. As an autogenous tissue, bone graft is a better option but is unsatisfactory due to its variable resorption and difficult handling properties [16, 17]. For most surgeons, an autogenous cartilage graft is the first choice in rhinoplasty because of its resistance to resorption and infection [18]. The common choices of autogenous cartilage graft in rhinoplasty are septal cartilage, auricular cartilage, or rib cartilage [19–21]. In cleft lip nasal deformity among Oriental patients, additional structural support is required to achieve the correction due to weak lower lateral cartilages [22]. Here, we had chosen rib cartilage as a source of graft in our cleft lip rhinoplasty. We described our technique of augmentation using L-shaped cartilage strut implant to improve the appearance of cleft lip-associated nose among Oriental patients and the outcome was evaluated according to patients' own perception. This was based on the premise that aesthetic technique in nasal augmentation with rib cartilage graft can provide perceivable and satisfactory improvement in patients with cleft lip nasal deformity.

## 2. Materials and Methods

A total of 16 patients, eleven females and five males, with nonsyndromic cleft lip and palate, were identified through retrospective chart review. All patients underwent augmentative open cleft lip rhinoplasty with L-shaped rib cartilage strut implant. Six of the patients were of Chinese ethnicity and 10 Malay. The ages of patients ranged from 14 to 33 years with the median and mean age at 18.5 and 20.4 years, respectively. Nine patients have left unilateral cleft lip and palate (UCLP) deformity, 3 have right UCLP, and 4 with bilateral cleft lip and palate (BCLP) deformity. All patients had clinical follow-up for more than 18 months postoperatively. The patients were interviewed using standard questionnaire in the clinic or by phone (Table 1). The questionnaire was used for the patients to rate their own perceived preoperative appearance and postoperative outcomes and their overall satisfaction with this technique. This is particularly important because it allowed us to assess our technique based on the patients' perception.

### 2.1. Operative Technique

A marginal (infracartilaginous) incision was made in both nostrils and continued in the columella by a transcolumellar, stair-step incision. This open approach allowed visualization of the cartilaginous and bony vault to facilitate accurate dissection of nasal pocket overlying the lower lateral cartilages, septal cartilage, and nasal bones. The columella and nasal skin flap were raised at supraperichondrial and supraperiosteal level without interfering with the underlying perichondrium and periosteum, respectively. The size of nasal pocket dissection was determined by intraoperative appearance of desired nasal augmentation. It is important to avoid overzealous dissection that can result in unstable cartilage graft position. Care was taken to avoid soft tissue irregularities by judiciously smoothening the undersurface of the soft tissue envelope to prevent overlying irregularities.

**Figure 1:**
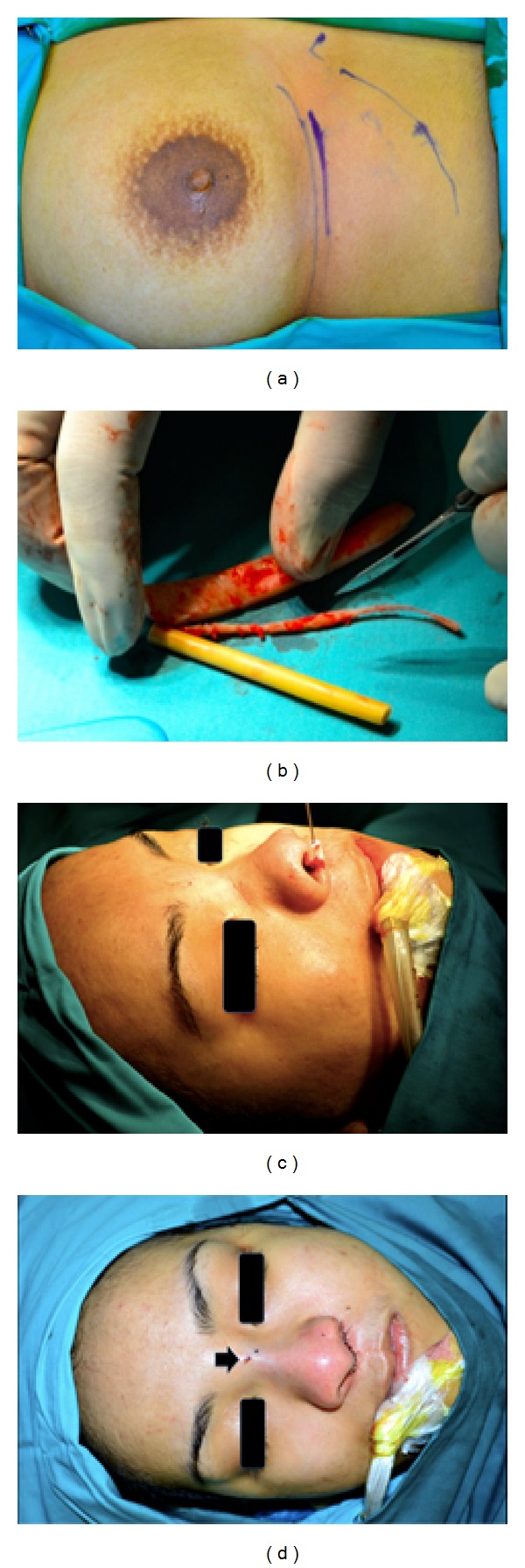
(a) Diagram showing the skin marking of the submammary incision. The curved line drawn inferior to the submammary marking represented the inferior margin of the rib cage. (b) The fabrication process of the harvested rib cartilage in which equal portion of the cartilage peripheries was shaved such that only the central most portion of the cartilage was retained for grafting. (c) The columellar component of the L-shaped rib cartilage was secured to the nasal spine, just inferior to the medial crus of lower lateral cartilages with 0.8 mm sized K-wire. (d) The proximal end of the dorsal onlay graft was secured to the nasal bone using K-wire (black arrow).

Rib cartilage was harvested from the 6th rib through submammary incision in female and subcostal incision in male (Figure 1(a)). The technique in rib cartilage graft harvest has been well described by Marin et al. [23]. The harvested rib cartilage was stripped off its peripheral portion such that only the central portion was utilized for fabrication and implant (Figure 1(b)). The 7 cm harvested cartilage graft was carved to appropriate size and shape and contoured with number 10 blade to allow accurate fashioning before implantation. Two components, the dorsal onlay and columellar component of the L-shaped cartilage strut, were obtained from the harvested rib cartilage. The dorsal onlay component is approximately 6 cm in length and columellar component 1 cm. The length of the graft had to be tailored to all individual cases. The grafts were then placed in position to improve cleft lip nose appearance through augmentation of the nasal dorsum and tip support. Finer fabrication proceeded carefully from this point usually by scraping the grafts with the sharp edge of a number 10 blade perpendicular to the graft surface until the exact desired size, shape, and contour were obtained. Note that it is important to tailor the size of the L-shaped cartilage strut to allow nasal augmentation that is proportionate to the facial aesthetic of patient.

After the final position was determined, K-wire was used to anchor the graft in position. The columellar component of the L-shaped rib cartilage was placed just inferior to the medial crus of lower lateral cartilages and secured with 0.8 mm sized smooth K-wire to the nasal spine (Figure 1(c)). The dorsal onlay component of the L-shaped cartilage strut was then inserted to the dorsal nasal pocket with its tip overlying the columellar component at the nasal tip. The position of the dorsal onlay graft was secured to the nasal bone using K-wire and covered with rubber tip from syringe plunger (Figure 1(d)). Note that a small pinhole was created only on the undersurface of the dorsal onlay graft over the columellar component to allow the fitting of the K-wire tip from the nasal spine into the pinhole. The percutaneous K-wire over the nasal bone was removed in the office with a wire twister one week postoperatively when the external splint was also removed.

## 3. Results 

In our series of patients with cleft lip nasal deformity, 87.5% (14 out of 16 patients) of patients perceived nasal asymmetry as the most undesirable aspect of a cleft lip nose (Table 2). This was followed by deformity associated with the nasal alae in which 12 out of 16 patients (75%) perceived the alar deformity as severe. Thirteen patients out of the 16 rated nasal tip deformity as moderate to severe. Majority of the patients rated the dorsum of nose deformity as mild and no patients perceived nasal apertures deformity as severe.

After augmentative open cleft lip rhinoplasty, 12 out of 16 (75%) patients reported good or excellent improvement in their nasal symmetry (Table 3). Thirteen patients rated satisfactory or good outcome on the appearance of nasal alae after operation. All patients reported good outcome on nasal tip projection and excellent result for dorsum of nose. The improvement to nasal apertures was unremarkable in 6 patients with 10 others experiencing satisfactory to good outcomes.

There were 3 complications in our series. Two patients with left UCLP experienced L-shaped cartilage graft displacement that required revision surgery. Both cases involved the displacement of the proximal part of the dorsal onlay component due to exceedingly large dorsal nasal pocket. One patient with BCLP had minor wound dehiscence over the columella. The wound dehiscence settled uneventfully with conservative management.

**Figure 2:**
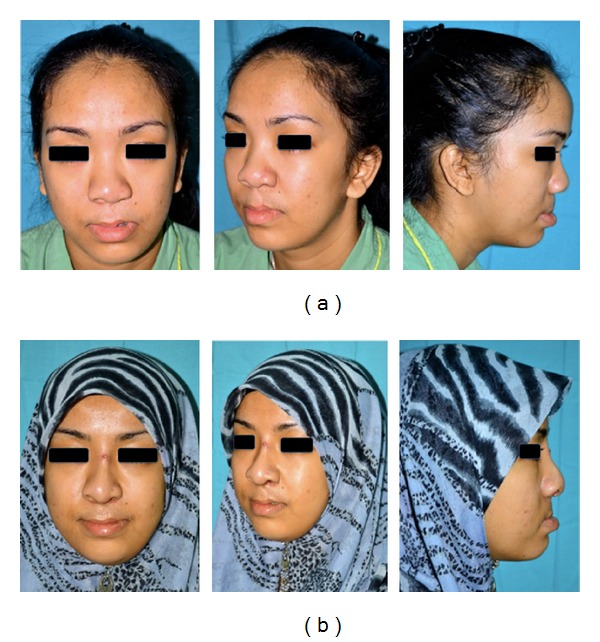
A 24-year-old lady with left unilateral cleft lip and palate. The photographs on the top row were taken preoperatively and those on the bottom row postoperatively. Her main complaints were asymmetrical nose with flat dorsum and broad left alar base on the cleft side. Postoperatively, her nasal symmetry had improved with increased dorsal nasal height and tip projection.

Although there was no rib cartilage graft donor site complication, two patients reported moderate degree of discomfort on the donor site. Majority of the patients (12 patients out of 16) experienced mild discomfort on the donor site during the immediate postoperative period. Other two expressed no discomfort from the donor site. Although a total of 14 patients (87.5%) experienced mild or moderate discomfort during the immediate postoperative period, 93.7% of them scored a visual analogue score (VAS) outcome satisfaction of 5–8 in which 0 represented the worst overall experience and 10 the most pleasant. All patients would recommend the procedure to a friend based on their own experience (Figure 2).

## 4. Discussion

In clinical practice, there is no universal concept of the “perfect face,” and there is not a specific shape of the nose, considered a model of beauty [1]. The definition of nasal aesthetics varies between different cultures and ethnicity in addition to the influence by popular trends of the society [8, 24]. Previous studies comparing the morphology of the Oriental and Caucasian nose noted remarkable differences between them [2, 7]. In Oriental patients, the nasal tip is bulbous with broad alar bases and lacking nasal height and tip projection [9, 11]. Although the deformity in cleft lip nasal deformity varied in its severity, it is characterized by nose that is asymmetrical with a similarly flat dorsum, broad tip, and wide alar base at the cleft side [5, 10]. According to Aung et al., they found that most Oriental patients prefer to have a higher nasal dorsum, increased nasal tip projection, and less flaring of the alar bases [9]. This same, desired nasal feature is also requested among patients with cleft lip nasal deformity [10]. In our group of patients, they rated nasal asymmetry, nasal alae, and tip as the most undesirable anatomical sites of cleft lip-associated nose.

The characteristic cleft lip nose represents a stigma to the cleft lip patient [25]. It is important for these patients to receive not only anthropometric normalization but also aesthetic improvement of the external nose to camouflage the patient's facial deformity [26]. In our series of patients, we used augmentation rhinoplasty technique to correct the nose of patients with cleft lip nasal deformity. We applied L-shaped cartilage strut to augment the nasal profile of our patients with cleft lip nasal deformity. Majority of our patients perceived this as a viable technique to improve their nasal appearance and had rated satisfactory to excellent outcomes for nasal symmetry, nasal alae, and tip of nose appearance after operation. The dorsal onlay component of the L-shaped strut helped to augment the nasal bridge, thus giving the illusion of a narrower and longer nose. There was, however, less improvement noted on the nasal apertures in our series of patients. Six patients rated unremarkable changes on their nasal apertures after operation. This was because augmentative procedure alone only altered the dimension of the nasal apertures and not its size.

In severely deformed, Oriental cleft lip-associated nose, the naturally thick overlying skin, bulbous nasal tip, and weak lower lateral cartilages among Orientals warrant additional structural support to achieve and maintain their correction [11, 27]. Here, we incorporated the columellar strut to enhance the definition and projection of the nasal tip and stabilized the caudal end of the dorsal onlay nasal strut on top of the columellar strut. The overall effect of the augmentation improved nasal symmetry and profile. This was consistent with studies by Yonehara et al. [10], and Jin and Won [24]. It should also be noted that augmentation with both dorsal nasal onlay grafts and columellar struts is not always performed in combination if only one is needed [27]. According to Yonehara et al., cantilever iliac bone graft alone was used but they experienced some loss of nasal tip definition in their patients [10].

In our series of 16 patients, we harvested rib cartilage graft as our cartilage donor. There was no complication with the donor site other than tolerable degree of discomfort in immediate postoperation period. Rib cartilage graft was chosen because it offered abundant supply and the strength needed for our L-shaped strut. Rib cartilage has been recognized to be the most reliable cartilage when structural support is needed in rhinoplasty [20, 21]. It is considerably versatile with respect to shape, length, and width that is important to our fabrication. In augmentation of cleft lip rhinoplasty, it is essential that each fabrication of the L-shaped cartilage strut implant was tailored to the individual needs of the patient [24]. To achieve consistent and satisfactory long-term outcomes, it is important to use rib cartilage graft for its low resorption rate and strength of support [20, 21, 24].

One of the issues associated with cartilage graft is the risk of warping [21, 24]. We did not notice warping among our patients in 18-month follow-up because only the central portion of the rib cartilage was used in our fabrication. It is also important to note that, during cartilage contouring, it has to be carried out in symmetry, meaning that equal portion of the cartilage external surface was discarded (Figure 1(b)). Harris et al. had shown that there was less risk of warping in the central portion of the cartilage [28].

One of our complications was the displacement of dorsal onlay component of the L-shaped strut in two of our patients. In both cases, the pocket that was created for the placement of the dorsal onlay graft was too large. It should be noted that the dorsal nasal pocket dissected should just accommodate the implant. It was possible that, with open rhinoplasty approach, overzealous visualisation and dissection may have resulted in the creation of the excessively large dorsal nasal pocket in both cases.

Another complication was in a patient with BCLP in which she sustained wound dehiscence over the transcolumellar incision site. The wound dehiscence occurred due to inherent short columella in BCLP and was further aggravated by the use of a sturdy columellar component of the L-shaped strut that forced a tight closure.

In our case series, the L-shaped rib cartilage strut continued to show good result at 18-month follow-up. This was consistent with the study by Yilmaz et al., which showed satisfactory nasal profile using rib cartilage grafts for dorsal nasal augmentation [29]. They found that the resorption rates were not high enough to change the shape of their augmented nose at 2-year follow-up. This is particularly important to provide cleft lip nasal deformity patients with a stable and long-term improvement. Our patients continued to be satisfied with the outcome of our cleft lip rhinoplasty that they would recommend their friends to undertake the same procedure.

With the majority of patients who reported good to excellent improvement in their nasal symmetry and profile, L-shaped strut augmentation rhinoplasty with rib cartilage graft presented a viable option in cleft lip rhinoplasty. Using this technique, we were successful in providing our cleft lip nose patients with a perceivable and satisfactory improvement to their nasal deformity.

## 5. Conclusion

Augmentation rhinoplasty in cleft lip nose can provide patients with a stable and satisfactory nasal appearance. This technique of using L-shaped rib cartilage strut provides many surgeons with added option in their quest to improve the appearance of patients with cleft lip nose. In conclusion, augmentation of cleft lip rhinoplasty can be employed with satisfactory outcome among Oriental patients with cleft lip nasal deformity.

## Figures and Tables

**Table 1:** Questionnaire on patients' perception of their cleft lip nasal deformity before and after operation and overall satisfaction of the procedure.

Questionnaire: cleft lip nasal deformity before and after operation and overall satisfaction					
The most undesirable anatomical sites before operation	None	Mild	Moderate	Severe	Unbearable
Nasal symmetry					
Nasal tip					
Dorsum of nose					
Nasal alae					
Nasal apertures					
Rib graft donor site discomfort	None	Mild	Moderate	Severe	Unbearable
The most improved anatomical sites after operation	Worse	Unremarkable	Satisfactory	Good	Excellent
Nasal symmetry					
Nasal tip					
Dorsum of nose					
Nasal alae					
Nasal apertures					
Overall satisfaction of procedure (VAS 0–10)					
VAS score					
Would you recommend it to a friend?	Yes	No			

**Table 2:** The most undesirable anatomical sites before operation as perceived by patients.

The most undesirable anatomical sites before operation	None	Mild	Moderate	Severe	Unbearable
Nasal symmetry			2	14	
Nasal tip		3	8	5	
Dorsum of nose		11	2	2	
Nasal alae		1	3	12	
Nasal apertures		12	4		

**Table 3:** The most improved anatomical sites after operation as perceived by patients.

The most improved anatomical sites after operation	Worse	Unremarkable	Satisfactory	Good	Excellent
Nasal symmetry			4	11	1
Nasal tip				16	
Dorsum of nose					16
Nasal alae		3	7	6	
Nasal apertures		6	8	2	

## References

[1] Szychta P, Rykała J, Kruk-Jeromin J (2011). Individual and ethnic aspects of preoperative planning for posttraumatic rhinoplasty. *European Journal of Plastic Surgery*.

[2] Cho BC, Baik BS (2001). Correction of cleft lip nasal deformity in Orientals using a refined reverse-U incision and V-Y plasty. *British Journal of Plastic Surgery*.

[3] Van Beek AL, Hatfield AS, Schnepf E (2004). Cleft rhinoplasty. *Plastic and Reconstructive Surgery*.

[4] Dibbell DG (1982). Cleft lip nasal reconstruction: correcting the classic unilateral defect. *Plastic and Reconstructive Surgery*.

[5] van Loon B, Maal TJ, Plooij JM (2010). 3D Stereophotogrammetric assessment of pre- and postoperative volumetric changes in the cleft lip and palate nose. *International Journal of Oral and Maxillofacial Surgery*.

[6] Foda HMT, Bassyouni K (2000). Rhinoplasty in unilateral cleft lip nasal deformity. *Journal of Laryngology and Otology*.

[7] Dong Y, Zhao Y, Bai S, Wu G, Wang B (2010). Three-dimensional anthropometric analysis of the Chinese nose. *Journal of Plastic, Reconstructive and Aesthetic Surgery*.

[8] Patel SM, Daniel RK (2012). Indian American rhinoplasty: an emerging ethnic group. *Plastic and Reconstructive Surgery*.

[9] Aung SC, Foo CL, Lee ST (2000). Three dimensional laser scan assessment of the Oriental nose with a new classification of Oriental nasal types. *British Journal of Plastic Surgery*.

[10] Yonehara Y, Takato T, Matsumoto S, Mori Y, Nakatsuka T, Hikiji H (2000). Correction of cleft ltp nasal deformity in orientals with a cantilevered iliac bone graft. *Scandinavian Journal of Plastic and Reconstructive Surgery and Hand Surgery*.

[11] Jin H, Won T (2009). Nasal tip augmentation in Asians using autogenous cartilage. *Otolaryngology—Head and Neck Surgery*.

[12] Hiraga Y (1980). Complications of augmentation rhinoplasty in the Japanese. *Annals of Plastic Surgery*.

[13] Raghavan U, Jones NS, Romo T (2004). Immediate autogenous cartilage grafts in rhinoplasty after alloplastic implant rejection. *Archives of Facial Plastic Surgery*.

[14] Godin MS, Waldman SR, Johnson CM, Stucker FJ (1995). The use of expanded polytetrafluoroethylene (Gore-Tex) in rhinoplasty. A 6-year experience. *Archives of Otolaryngology—Head and Neck Surgery*.

[15] Davis PKB, Jones SM (1971). The complications of silastic implants. Experience with 137 consecutive cases. *British Journal of Plastic Surgery*.

[16] Chase SW, Herndon CH (1955). The fate of autogenous and homogenous bone grafts. *The Journal of Bone and Joint Surgery A*.

[17] Mowlem R (1963). Bone grafting. *British Journal of Plastic Surgery*.

[18] Tosun Z, Karabekmez FE, Keskin M, Duymaz A, Savaci N (2008). Allogenous cartilage graft versus autogenous cartilage graft in augmentation rhinoplasty: a decade of clinical experience. *Aesthetic Plastic Surgery*.

[19] Matsuo K, Hirose T (1990). Secondary correction of the unilateral cleft lip nose using a conchal composite graft. *Plastic and Reconstructive Surgery*.

[20] Gunter JP, Rohrich RJ (1990). Augmentation rhinoplasty: dorsal onlay grafting using shaped autogenous septal cartilage. *Plastic and Reconstructive Surgery*.

[21] Gunter JP, Clark CP, Friedman RM (1997). Internal stabilization of autogenous rib cartilage grafts in rhinoplasty: a barrier to cartilage warping. *Plastic and Reconstructive Surgery*.

[22] Jang T, Choi Y, Jung Y, Kim K, Kim K, Choi J (2007). Effect of nasal tip surgery on Asian noses using the transdomal suture technique. *Aesthetic Plastic Surgery*.

[23] Marin VP, Landecker A, Gunter JP (2008). Harvesting rib cartilage grafts for secondary rhinoplasty. *Plastic and Reconstructive Surgery*.

[24] Jin HR, Won TB (2011). Recent advances in Asian rhinoplasty. *Auris Nasus Larynx*.

[25] Chaithanyaa N, Rai KK, Shivakumar HR, Upasi A (2011). Evaluation of the outcome of secondary rhinoplasty in cleft lip and palate patients. *Journal of Plastic, Reconstructive and Aesthetic Surgery*.

[26] Rikimaru H, Kiyokawa K, Koga N, Takahashi N, Morinaga K, Ino K (2008). A new modified forked flap with subcutaneous pedicles for adult cases of bilateral cleft lip nasal deformity: from normalization to aesthetic improvement. *The Journal of Craniofacial Surgery*.

[27] Takato T, Yonehara Y, Mori Y, Susami T (1995). Use of cantilever iliac bone grafts for reconstruction of cleft lip-associated nasal deformities. *Journal of Oral and Maxillofacial Surgery*.

[28] Harris S, Pan Y, Peterson R, Stal S, Spira M (1993). Cartilage warping: an experimental model. *Plastic and Reconstructive Surgery*.

[29] Yilmaz M, Vayvada H, Menderes A, Mola F, Atabey A (2007). Dorsal nasal augmentation with rib cartilage graft: long-term results and patient satisfaction. *Journal of Craniofacial Surgery*.

